# Supplementary material for: Potential effects of metal ion induced two-state allostery on the regulatory mechanism of add adenine riboswitch
Source: Commun Biol. 2022 Oct 22;5:1120. doi: 10.1038/s42003-022-04096-z (PMC9588036; doi:10.1038/s42003-022-04096-z)
Supplement: Supplementary file 2 — Supplementary Information [file 42003_2022_4096_MOESM2_ESM.pdf]

Supplementary Information

Potential effects of metal ion induced two-state allostery on the regulatory mechanism of *add* adenine riboswitch

Lei Bao[1,*], Wen-Bin Kang[1], and Yi Xiao[2]

[1]School of Public Health, Hubei University of Medicine, Shiyan, Hubei 442000, China

[2]Institute of Biophysics, School of Physics, Huazhong University of Science and Technology, Wuhan, Hubei 430074, China

[*]Correspondence and requests for materials should be addressed to L.B. (email: bolly@whu.edu.cn)

Supplementary Methods

S1. Determination of the π-π stacking between two bases

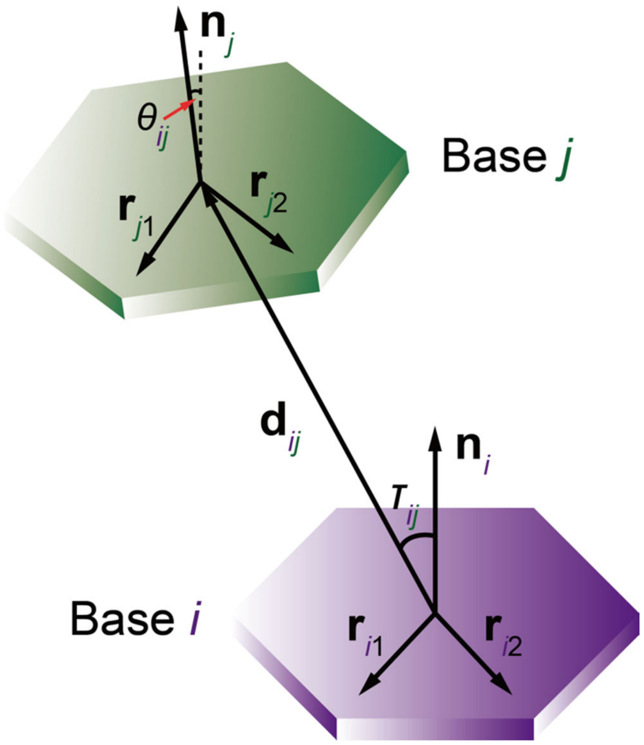

At first, a distance vector $\mathbf{d}_{ij}$ (cutoff of $||\mathbf{d}_{ij}|| = 4.0$ Å) connecting the geometric centers of two potentially stacked bases (i and j) is constructed (as shown in the figure above)[1]. For purines, the geometric centers of the pyrimidine rings are used instead for simplicity. Secondly, the mean plane of each ring is defined by two vectors $\mathbf{r}_1$ and $\mathbf{r}_2$. Then, the normal vectors for bases i and j are determined as:

$$\mathbf{n}_i = \frac{\mathbf{r}_{i1} \times \mathbf{r}_{i2}}{\left\| \mathbf{r}_{i1} \times \mathbf{r}_{i2} \right\|} \quad \text{(S1)}$$

$$\mathbf{n}_j = \frac{\mathbf{r}_{j1} \times \mathbf{r}_{j2}}{\left\| \mathbf{r}_{j1} \times \mathbf{r}_{j2} \right\|} \quad \text{(S2)}$$

If the angle between these two vectors (or its supplement) $\theta_{ij} = \cos^{-1}(\mathbf{n}_i \cdot \mathbf{n}_j)$ is less than the user-defined value, the overlap of the bases i and j is determined using the angle between the unit distance vector connecting the ring geometric centers and one of the base normal vectors:

$$\tau_{ij} = \cos^{-1}\left(\mathbf{n}_i \cdot \frac{\mathbf{d}_{ij}}{\left\| \mathbf{d}_{ij} \right\|}\right) \quad \text{(S3)}$$

If τ_{ij} (or its supplement) is less than the user-defined value, then the bases are considered stacked. If any of these criteria are not satisfied, the bases are assumed to be unstacked.

S2. Evaluate the motion correlation between any two residues

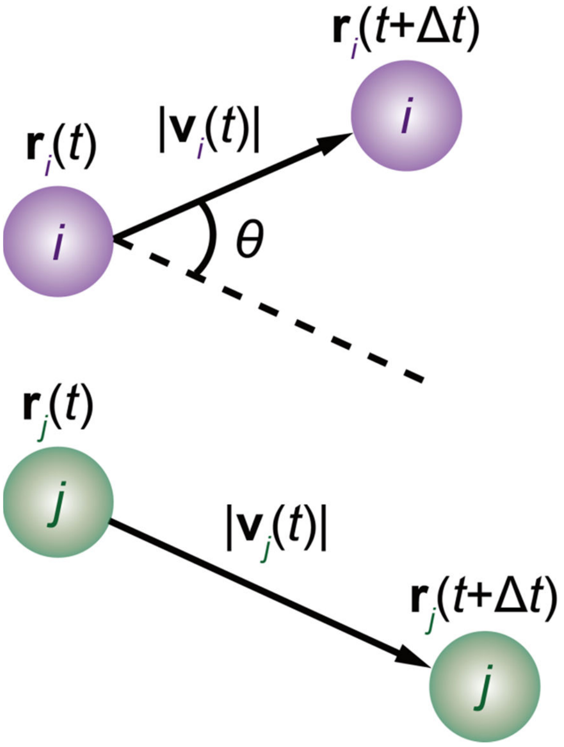

In this work, we use the velocity vector to measure the dynamic motion of residues. The velocity vector $\mathbf{v}(t)$ can be obtained by subtracting the position vector of the previous time $\mathbf{r}(t)$ from the position vector of the next time $\mathbf{r}(t+\Delta t)$:

$$\mathbf{v}(t) = \mathbf{r}(t+\Delta t) - \mathbf{r}(t) \tag{S4}$$

where Δt is the time interval. Thus, the smaller the time interval, the more accurate we describe the motions of residues.

It should be noted that velocity vector has two elements: direction and magnitude. Therefore, we used two ways to evaluate the motion correlation between any two residues (i and j). One is the correlation of motion direction, which can be expressed as the cosine value of angle between the velocity vectors of two residues:

$$\cos\theta = \frac{\mathbf{v}_i(t)\cdot\mathbf{v}_j(t)}{\left\|\mathbf{v}_i(t)\right\|\cdot\left\|\mathbf{v}_j(t)\right\|} \tag{S5}$$

another is the correlation between the magnitudes of velocity vectors:

$$r = \frac{\mathrm{cov}(\|\mathbf{v}_i(t)\|, \|\mathbf{v}_j(t)\|)}{\sigma(\|\mathbf{v}_i(t)\|) \cdot \sigma(\|\mathbf{v}_j(t)\|)} \quad \text{(S6)}$$

where $\mathrm{cov}(\|\mathbf{v}_i(t)\|,\|\mathbf{v}_j(t)\|)$ is the covariance between $\|\mathbf{v}_i(t)\|$ and $\|\mathbf{v}_j(t)\|$. $\sigma(\|\mathbf{v}_i(t)\|)$, and $\sigma(\|\mathbf{v}_j(t)\|)$ are the standard deviations of $\|\mathbf{v}_i(t)\|$ and $\|\mathbf{v}_j(t)\|$, respectively.

S3. Calculate the bulk concentration of metal ions

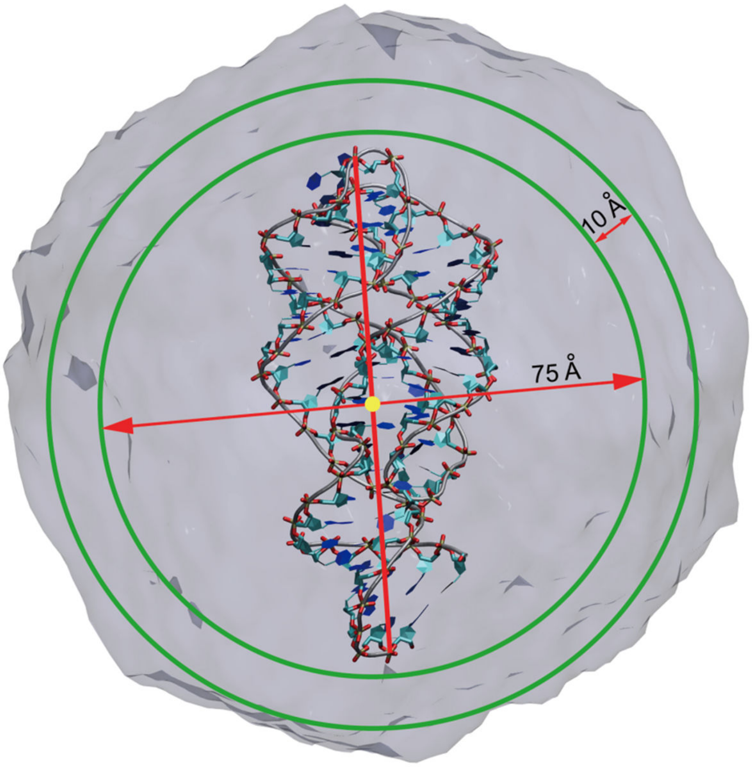

To calculate the bulk concentration of metal ions, we first determined the maximum distance between two residues of AARA in each frame. Then, these maximum distances were averaged over the simulation time. The results showed that the average value of maximum distance of AARA was below about 75 Å at different ionic conditions (Supplementary Table S1). Therefore, it can be considered that a sphere with a diameter of 75 Å can well wrap the whole AARA molecule.

In the next, we determined the midpoint of the line between the farthest two residues in each frame, and counted the number of metal ions n_i in the shell composed of two spheres with the diameter of 75 Å and 95 Å centered on the midpoint for each frame. Then, the number of metal ions was averaged over the simulation time. Finally, the approximate bulk

concentration C_{bulk} of metal ions can be obtained through dividing the average value of n_i (through a total of N conformations) by the volume of spherical shell V:

$$C_{\text{bulk}} = \frac{1}{NV}\sum_{i=1}^{N} n_i \tag{S7}$$

S4. Calculate the scattering patterns from MD trajectories

Small angle X-ray scattering (SAXS) is a method that can effectively probe the shape and structure of biomolecules. In experiments, X-ray scattering on biomolecules usually compare the scattering intensity from a biomolecule solution (system A) to a "blank" containing just pure solvent (system B), and report the difference, or "excess" intensity:

$$I(\mathbf{q}) = \left\langle |A(\mathbf{q})|^2 \right\rangle_t - \left\langle |B(\mathbf{q})|^2 \right\rangle_t \tag{S8}$$

where the $\langle\ \rangle_t$ bracket indicates the intensities are averaged over the measurement time and volume. $\mathbf{q}$ is the scattering wave vector, and the magnitude of the scattering wave vector q is related to the scattering angle 2θ by $q = (4\pi/\lambda)\sin\theta$, where λ is the wavelength of the incident X-ray beam.

The total intensity can be approximately rewritten as[2,3]:

$$I(\mathbf{q}) = \left[\langle A_1(\mathbf{q})\rangle - \langle B_1(\mathbf{q})\rangle\right]^2 + \left[\left\langle |A_1(\mathbf{q})|^2 \right\rangle - \left|\langle A_1(\mathbf{q})\rangle\right|^2\right] - \left[\left\langle |B_1(\mathbf{q})|^2 \right\rangle - \left|\langle B_1(\mathbf{q})\rangle\right|^2\right] \tag{S9}$$

where $A_1(\mathbf{q})$ and $B_1(\mathbf{q})$ are Fourier transforms for system A and B but only considering regions where there is excess electron density relative to the bulk value.

For MD simulation, the intensity can be further rewritten as[2]:

$$I(\mathbf{q}) = |a(\mathbf{q}) - b(\mathbf{q})|^2 + \frac{1}{N}\sum_i \left|A_1^{(i)}(\mathbf{q}) - a(\mathbf{q})\right|^2 - \frac{N'+1}{N'(N'-1)}\sum_j \left|B_1^{(j)}(\mathbf{q}) - b(\mathbf{q})\right|^2 \tag{S10}$$

where $A_1^{(i)}(\mathbf{q})$ and $B_1^{(i)}(\mathbf{q})$ are the scattering amplitudes of the each snapshot from the system A and system B, respectively, and can be computed by:

$$A_1(\mathbf{q}) = \sum_n f_n(\mathbf{q}) e^{-i\mathbf{q}\cdot\mathbf{r}_n} \tag{S11}$$

where $\mathbf{r}_n$ is the coordinate of the nth atom contained in the biomolecule or the water shell of a selected thickness, and $f_n(\mathbf{q})$ represents the atomic form factors.

$a(\mathbf{q})$ and $b(\mathbf{q})$ are the averaged amplitudes for the total N and N' snapshots, respectively:

$$a(\mathbf{q}) = \frac{1}{N}\sum_{i=1}^{N} A_1^{(i)}(\mathbf{q}) \quad \text{(S12)}$$

$$b(\mathbf{q}) = \frac{1}{N'}\sum_{j=1}^{N'} B_1^{(j)}(\mathbf{q}) \quad \text{(S13)}$$

At last, rotating a biomolecule is equivalent to rotating the scattering wave vector **q** with respect to the biomolecule coordinates fixed in space. Therefore, after the rotational average is performed by using Lebedev quadrature, the total intensity can be obtained[3]:

$$I(q) = \frac{1}{4\pi}\int I(\mathbf{q})d\Omega \quad \text{(S14)}$$

In this work, we selected 100 evenly spaced frames along the whole production trajectories and used the built-in module saxs_md of Amber to estimate the total intensity. In detail, saxs_md takes input as two sets of coordinates extracted from snapshots of "sample" and "blank" MD simulations (the "sample" MD contains the biomolecule plus water and salt, while the "blank" MD simulations only has pure water plus salt). Here, the cutoff and spacing of q are 0.5 Å and 0.01 $Å^{-1}$, respectively.

In addition, the Kratky plot [$q^2I(q)$ as a function of q] also can be calculated directly from the scattering curve, which provide an excellent tool for evaluating the folding of samples. For folded biomolecules, the Kratky plot yields a peak roughly shaped like a parabola. In contrast, the extended or unfolded biomolecules lack this peak and have a plateau or are slightly increasing in the larger q-range.

Supplementary Figures

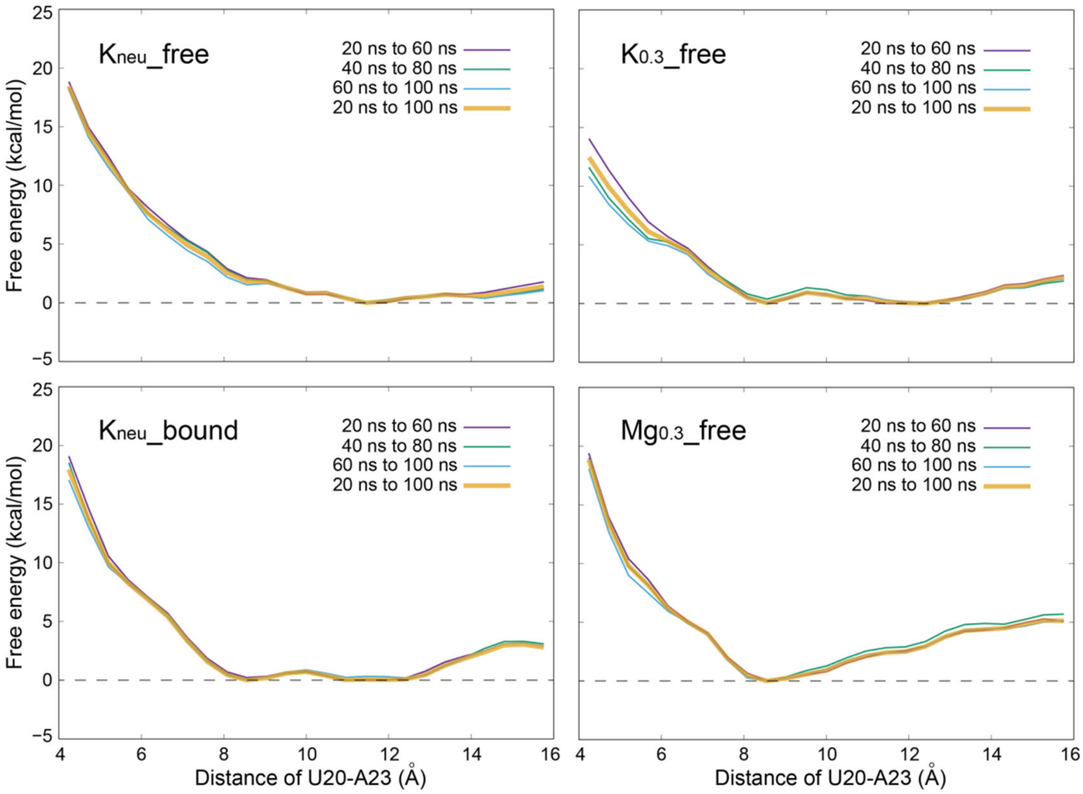

Fig. S1 To depict the convergence, the changes in PMF results from the umbrella sampling simulations through time are shown for all four ionic conditions.

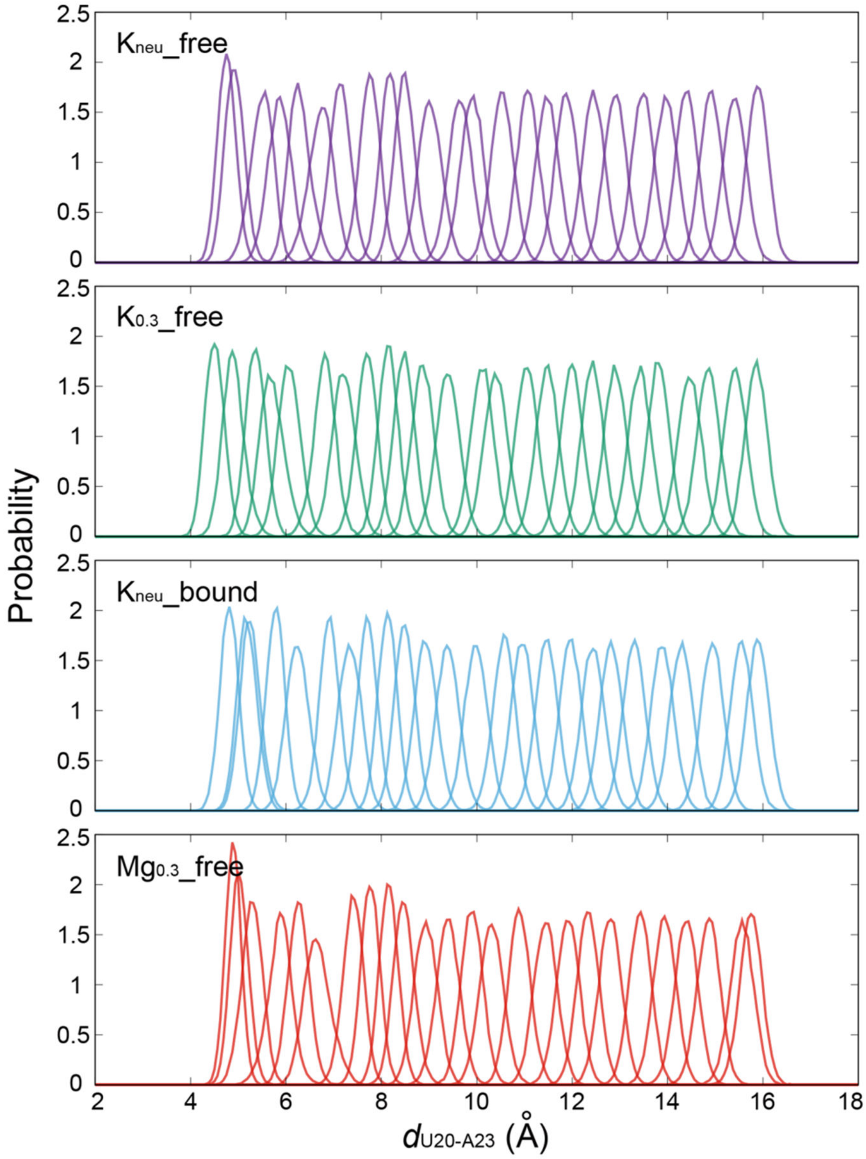

Fig. S2 The normalized distributions in all windows of umbrella sampling along the reaction coordinate $d_{U20\text{-}A23}$ at different ionic conditions. The initial 20 ns of sampling trajectories were disregarded, and only the last 80 ns of sampling data were used for WHAM calculation.

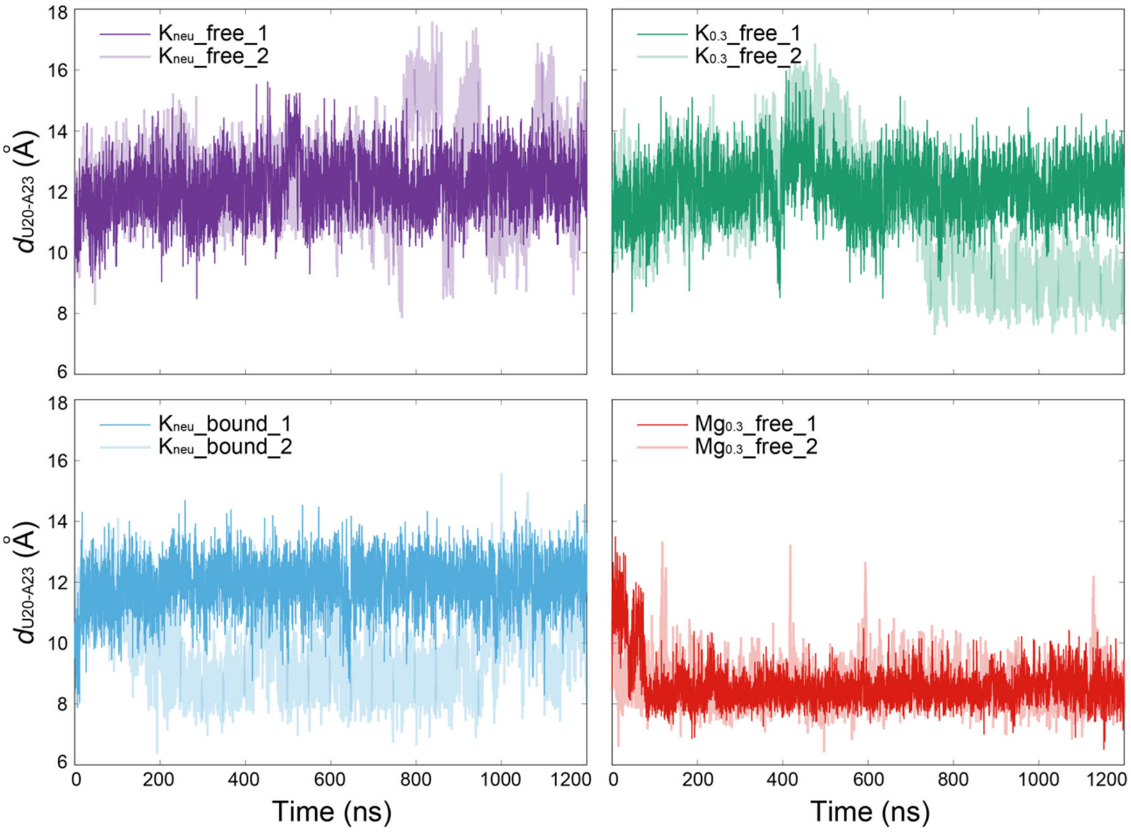

Fig. S3 The evolutions of $d_{U20\text{-}A23}$ versus MD time in all eight simulation trajectories. Obviously, there are transitions between two states in $K_{0.3}$_free_2 and K_{neu}_bound_2.

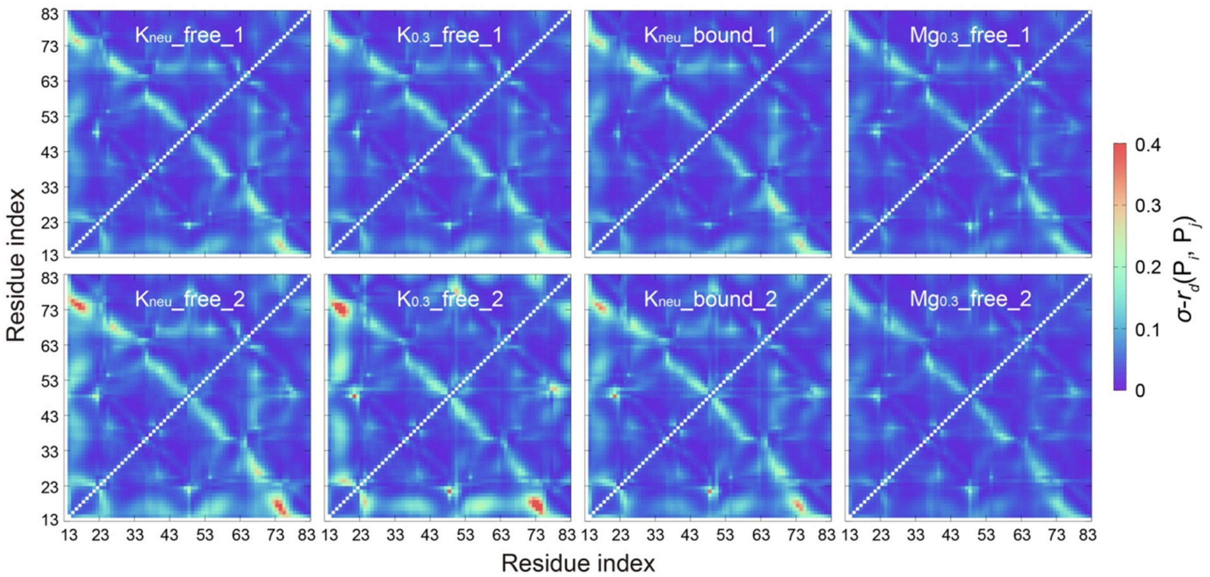

Fig. S4 The standard deviation of $r_{\Delta d}$(P$_i$, P$_j$) for all eight simulation trajectories. The color bar shows the variations in standard deviation of $r_{\Delta d}$(P$_i$, P$_j$) from 0 (dark blue) to 0.4 (red).

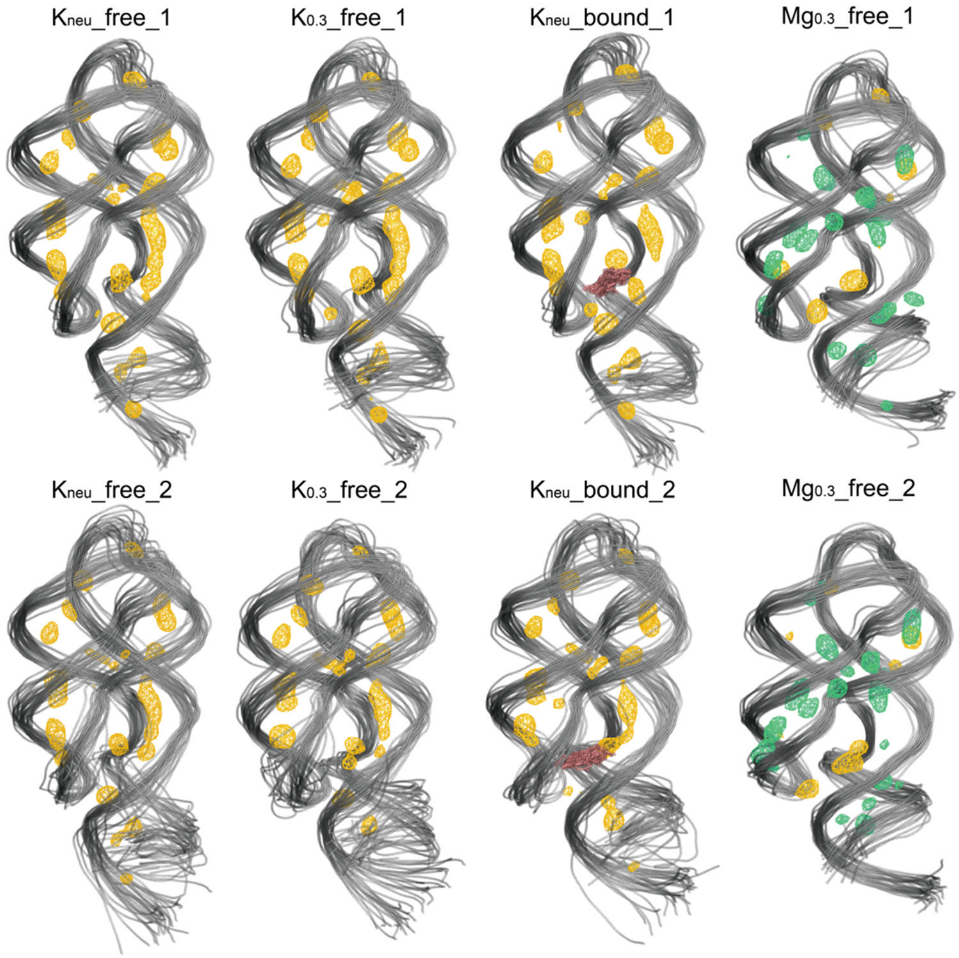

Fig. S5 The back view of the structure clusters of AARA and specific binding sites of metal ions around it in all eight simulation trajectories (all of 1.2 μs data). Here, the sampling interval for generating structure clusters is 40 ns (30 conformations in total). The specific binding sites are described by the spatial occupancies of metal ions with an isosurface of 0.1 Å^{-3} (K^+ in orange and Mg^{2+} in green). Here, the time-averaged spatial occupancies of metal ions were converted into density maps using the VolMap tool of the VMD molecular visualization program, and we chose a resolution of 0.5 Å^3.

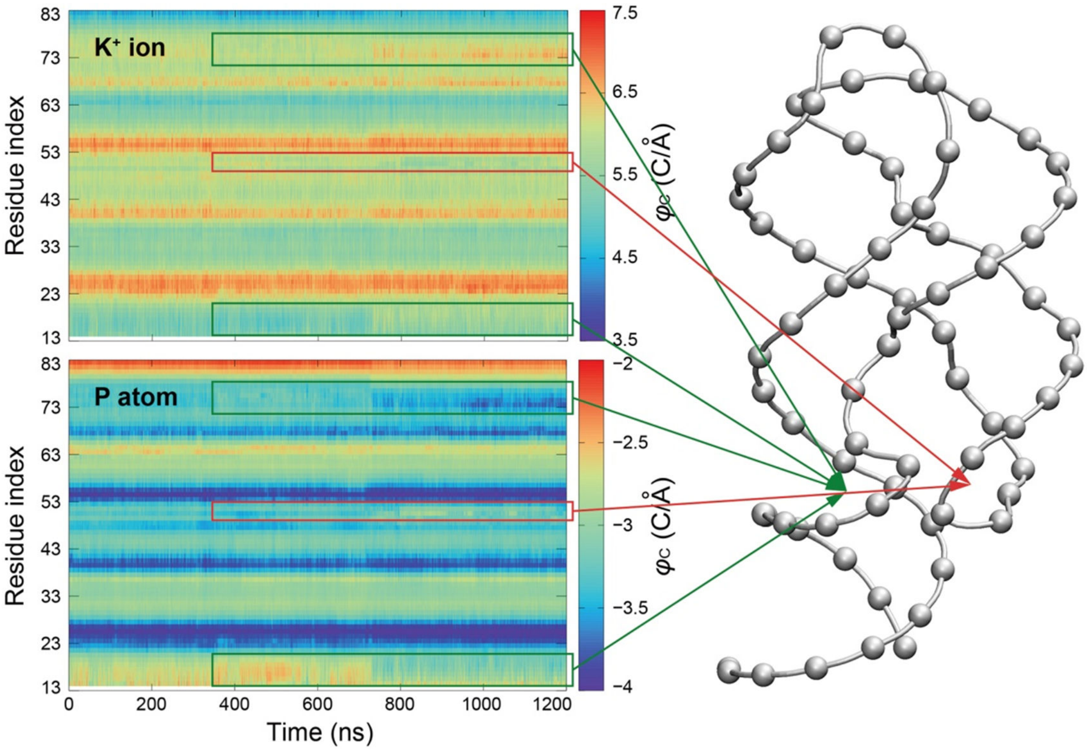

Fig. S6 The time evolutions of φ_C for K^+ ions (positively charged) and phosphate groups (negatively charged) at each residue position in $K_{0.3}$_free_2. Some regions (such as $C_{J12\text{-}P1}$ and J23) undergo large structural transition (accompanied with the change of electrostatic potential in these regions) and show strong correlation with the density of K^+ ion.

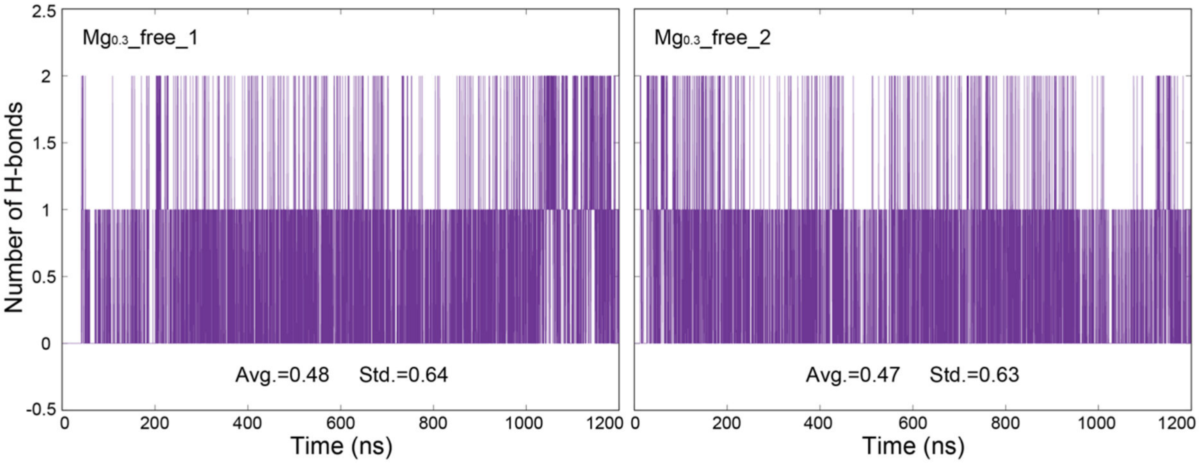

Fig. S7 The number of hydrogen bonds between U51 and U74 versus MD time in $Mg_{0.3}$_free_1 and $Mg_{0.3}$_free_2, and the average values (Avg.) and standard deviations (Std.) of the number of hydrogen bonds are shown in the figure.

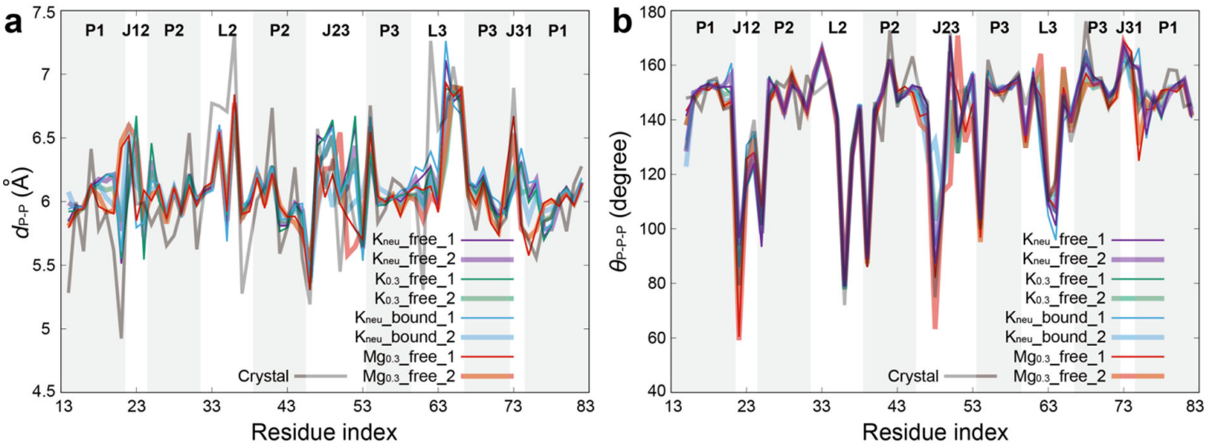

Fig. S8 **a** The distances between two phosphate groups along the backbone in all eight simulation trajectories and crystal structure. **b** The bending angles composed by three consecutive adjacent phosphate groups along the backbone in all eight simulation trajectories and crystal structure.

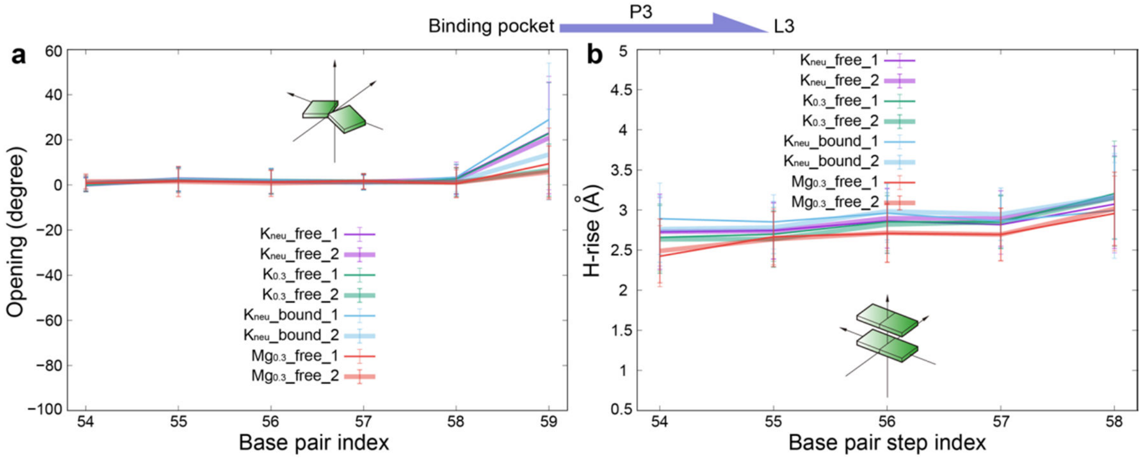

Fig. S9 The average values and standard deviations of Opening (**a**) and H-rise (**b**) of each base pair in P3 helix for all eight simulation trajectories. The direction of P3 (from binding pocket to L3) is also shown in the figure.

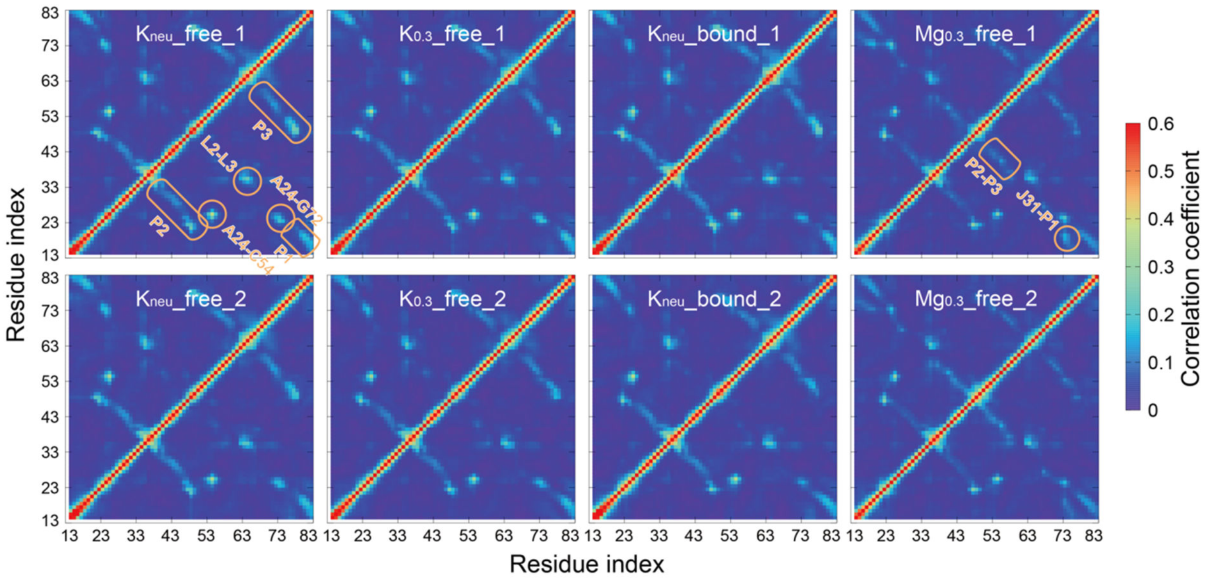

Fig. S10 The correlation maps of magnitude of velocity vector between any two phosphate groups for all eight simulation trajectories. The color bar shows the variations in correlation coefficient from 0 (dark blue) to 0.6 (red). Several regions showing significant correlation are marked with orange boxes.

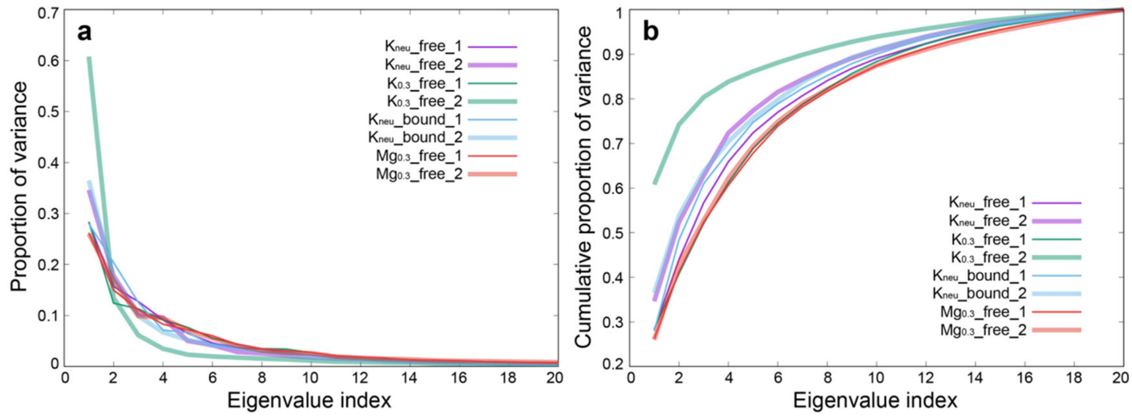

Fig. S11 a The proportion of variance described by the first 20 (eigenvalue index) principal components from PCA performed on the atomic displacement of phosphorus atoms on AARA for all eight simulation trajectories. **b** The cumulative proportion of variance described by the first *n* (eigenvalue index) principal components for all eight simulation trajectories.

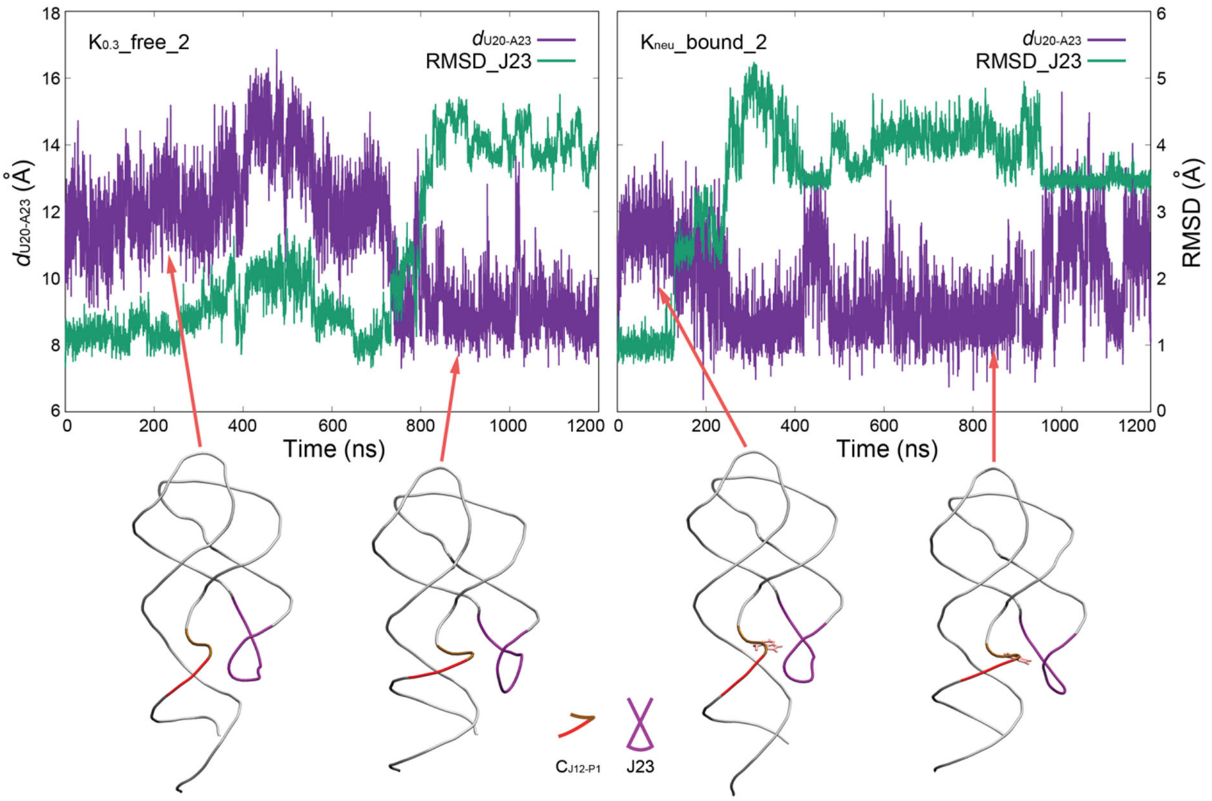

Fig. S12 The evolutions of $d_{U20\text{-}A23}$ (purple solid lines) and evolutions of RMSD of J23 (green solid lines) versus MD time in $K_{0.3}$_free_2 and K_{neu}_bound_2, and the structural correlation between $C_{J12\text{-}P1}$ (ochre and red) and J23 (purple) are also intuitively displayed in four typical snapshots.

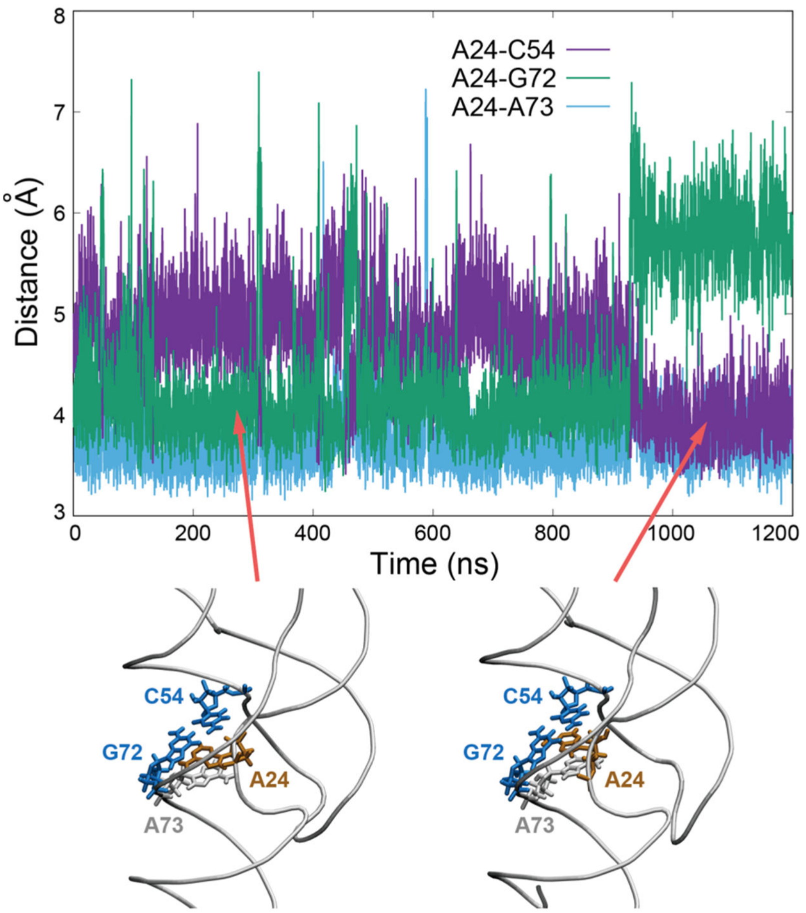

Fig. S13 The evolutions of distances between A24 and C54 (purple), between A24 and G72 (green), and between A24 and A73 (blue) versus MD time in $K_{0.3}$_free_2, and the relative positions of these residues in different time periods are visualized in two snapshots. For simplicity, the coordinates of bases are represented by the geometric center of the hexatomic ring they contain.

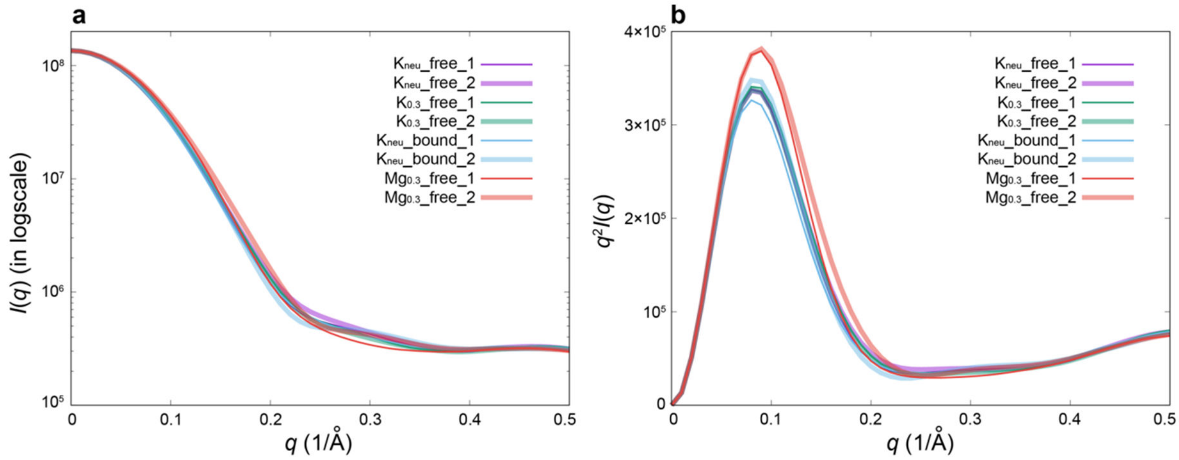

Fig. S14 a The SAXS profiles of AARA estimated from 100 evenly spaced frames along the whole production trajectories of all eight simulation trajectories. **b** The Kratky plots of the SAXS data of AARA in all eight simulation trajectories show asymmetric Bell-shape curves, characteristic of a folded structure with partial flexibility.

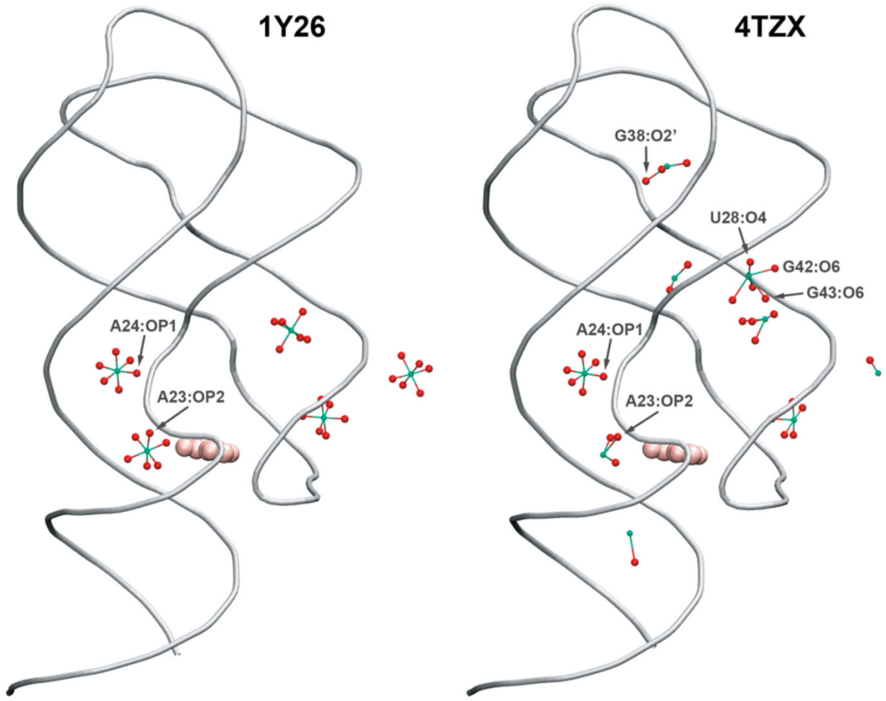

Fig. S15 Two crystal structures of AARA from X-ray diffraction experiments and their Protein Data Bank IDs are 1Y26[4] and 4TZX[5], respectively. The cognate ligand (pink), Mg^{2+} ions (green), and the oxygen atoms (red) of RNA and water molecules that interact with Mg^{2+} ions via inner-sphere contacts are shown with the van der Waals coloring method.

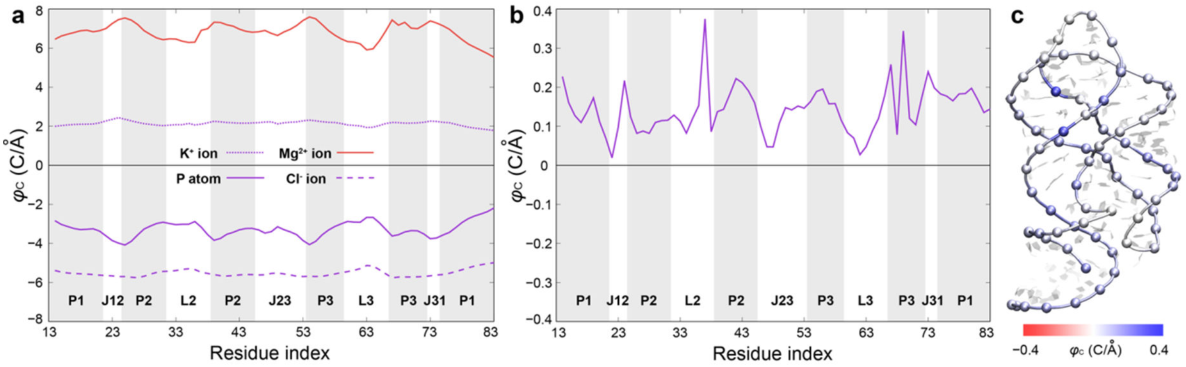

Fig. S16 a The cumulative electrostatic potential strength coefficients φ_C of phosphate group (purple solid line), K^+ ion (dense dashed line), Cl^- ion (sparse dashed line) and Mg^{2+} ion (red solid line) for $Mg_{0.3}$_free system (taking $Mg_{0.3}$_free_1 as an example) in different regions of AARA structure. **b** The total cumulative electrostatic potential strength coefficients φ_C of $Mg_{0.3}$_free system (taking $Mg_{0.3}$_free_1 as an example) in different regions of AARA structure. **c** The total cumulative electrostatic potential strength coefficients φ_C are shown in a typical structure from $Mg_{0.3}$_free_1, and the color bar shows the variations in φ_C from −0.4 C·Å^{-1} (red) to 0.4 C·Å^{-1} (blue).

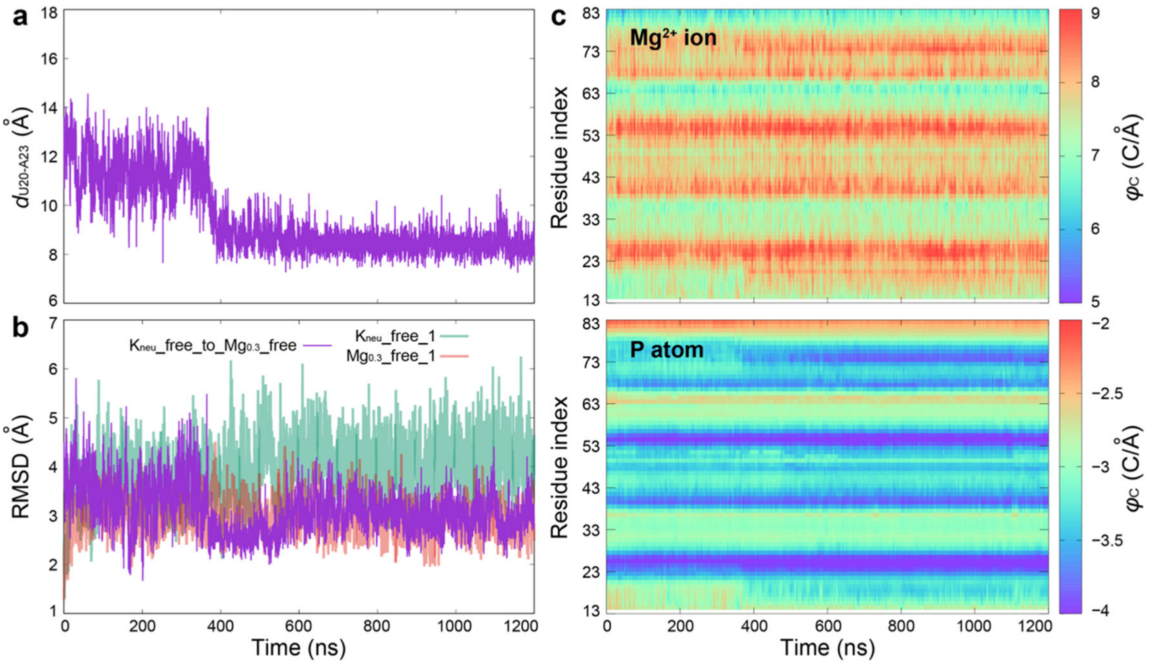

Fig. S17 a The time evolution of $d_{U20\text{-}A23}$ versus MD time in K_{neu}_free_to_$Mg_{0.3}$_free simulation trajectory. **b** The time evolutions of RMSD versus MD time in three simulation trajectories K_{neu}_free_to_$Mg_{0.3}$_free (purple), K_{neu}_free_1 (light green) and $Mg_{0.3}$_free_1 (light red). **c** The time evolutions of φ_C for Mg^{2+} ions (positively charged) and phosphate groups (negatively charged) at each residue position. In particular, $C_{J12\text{-}P1}$ undergo large structural transition (accompanied with the change of electrostatic potential in this region) and show strong correlation with the density of Mg^{2+} ion.

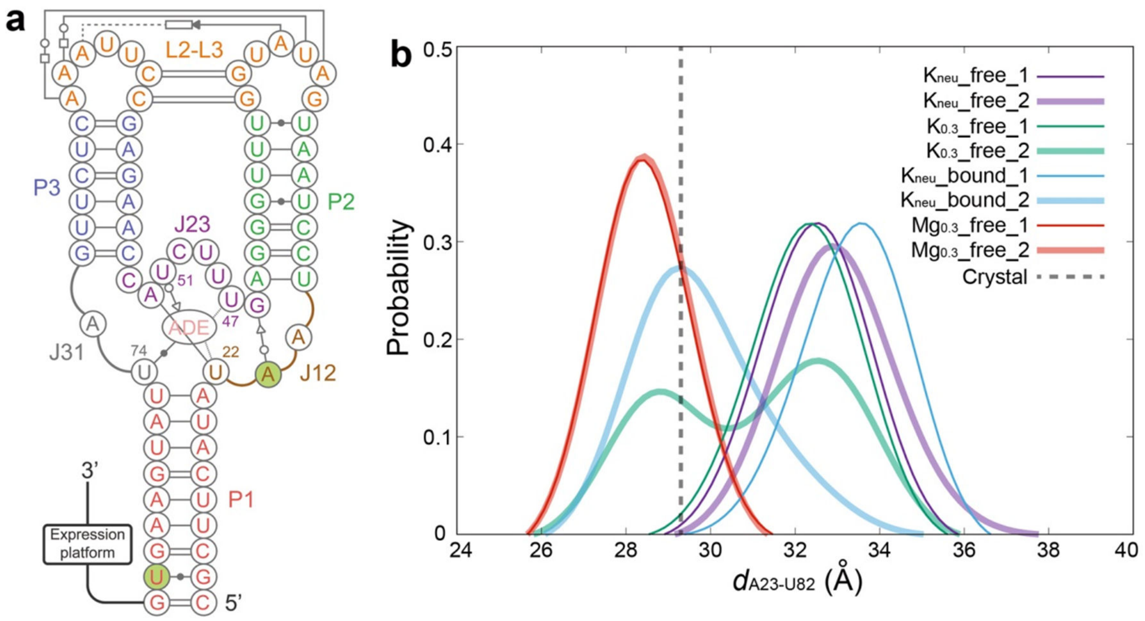

Fig. S18 a Schematic diagram of single-molecule FRET experiment design, the distance between A23 and U82 (filled with light green) may be suited for detection by applying fluorescence resonance effect. **b** The normalized distributions of $d_{\mathrm{A23\text{-}U82}}$ for all eight simulation trajectories, and the gray dashed line corresponds to $d_{\mathrm{A23\text{-}U82}}$ in the crystal structure (~29.3 Å).

Supplementary Tables

Table S1. The average values and standard deviations for the width of major groove (in Å) formed by helices P1 and P3 in all eight simulation trajectories. In the crystal structure (ligand-bound conformer), this width is about 16.6 Å[6].

	K_{neu}_free	$K_{0.3}$_free	K_{neu}_bound	$Mg_{0.3}$_free
Traj. 1	22.3 ± 1.9	21.6 ± 1.9	22.7 ± 2.0	14.0 ± 1.8
Traj. 2	23.7 ± 3.2	19.7 ± 4.6	18.1 ± 3.0	14.3 ± 1.9

Table S2. The stacking features between two relative residues (U74 and U75, A21 and A76) in two K_{neu}_bound trajectories (average value) and crystal structure. The schematic diagram of the interactions between corresponding residues is also shown below.

Residue index	d_{ij} (Å)			θ_{ij} (deg)			τ_{ij} (deg)		
	Crystal	K_{neu}_bound		Crystal	K_{neu}_bound		Crystal	K_{neu}_bound	
		Traj. 1	Traj. 2		Traj. 1	Traj. 2		Traj. 1	Traj. 2
i=74,j=75	3.9	5.1	4.1	9.5	37.5	15.8	27.0	35.8	31.7
i=21,j=76	3.7	3.8	3.8	4.0	9.7	10.1	27.7	29.4	29.4

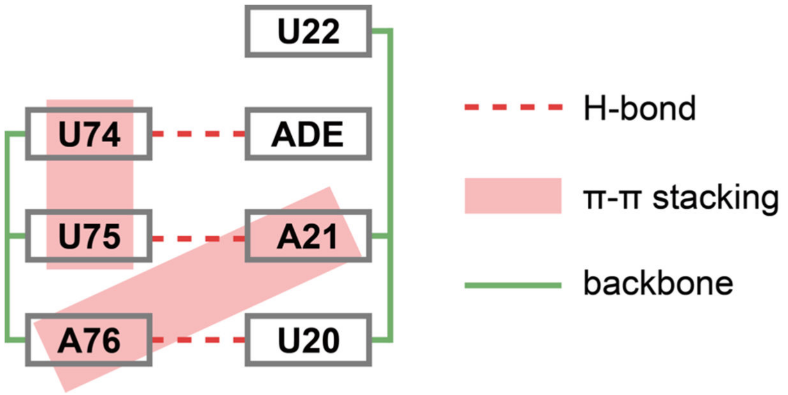

Table S3. The average values and standard deviations of SASA (in Å^2) of residues A21 and U75 (including only bases and excluding backbone parts) at different ionic conditions.

	K_{neu}_free	$K_{0.3}$_free	K_{neu}_bound	$Mg_{0.3}$_free
Traj. 1	133.6 ± 18.6	134.3 ± 25.9	132.2 ± 16.3	94.8 ± 16.8
Traj. 2	149.6 ± 30.9	139.0 ± 42.5	104.8 ± 11.8	113.8 ± 15.3

Table S4. The average values and standard deviations of interhelical angle (in degree) and interhelical distance (in Å) between P1 and P3 in all eight simulation trajectories and crystal structure.

	Crystal	K_{neu}_free		$K_{0.3}$_free		K_{neu}_bound		$Mg_{0.3}$_free	
		Traj. 1	Traj. 2	Traj. 1	Traj. 2	Traj. 1	Traj. 2	Traj. 1	Traj. 2
Interhelical angle (deg)	34.1	23.4 ± 8.9	24.5 ± 11.0	23.6 ± 9.1	27.6 ± 10.8	21.4 ± 8.8	34.1 ± 10.9	44.4 ± 9.3	40.0 ± 9.0
Interhelical distance (Å)	13.0	15.8 ± 1.3	17.4 ± 2.7	15.5 ± 1.3	15.0 ± 1.9	16.0 ± 1.2	14.1 ± 1.3	13.4 ± 0.9	13.3 ± 0.9

Table S5. The average values and standard deviations of the longest distance between residues with time, as well as the average values and standard deviations of the bulk concentrations of metal ions at different ionic conditions.

	K_{neu}_free		$K_{0.3}$_free		K_{neu}_bound		$Mg_{0.3}$_free	
	Traj. 1	Traj. 2	Traj. 1	Traj. 2	Traj. 1	Traj. 2	Traj. 1	Traj. 2
$\bar{d}_{max}$ (Å)	74 ± 2	75 ± 3	73 ± 2	71 ± 4	75 ± 2	69 ± 3	65 ± 2	66 ± 2
[K^+] (mM)	79 ± 19	79 ± 20	378 ± 48	372 ± 49	79 ± 19	72 ± 19	173 ± 29	176 ± 29
[Mg^{2+}] (mM)	—	—	—	—	—	—	225 ± 31	226 ± 30

Description of Supplementary Movies

File Name: Supplementary Movie 1

Description: The first principal component for $Mg_{0.3}$_free_1 shows that the Mg^{2+} ion bound at $C_{J12\text{-}P1}$ site fluctuates with the surrounding structure.

File Name: Supplementary Movie 2

Description: The rapid exchange phenomenon of Mg^{2+} ions at $C_{J12\text{-}P1}$ site in $Mg_{0.3}$_free_1. The chelated water molecules around Mg^{2+} ions and the structure near the binding site are depicted with licorice drawing method.

File Name: Supplementary Movie 3

Description: The rapid exchange phenomenon of K^{+} ions at the binding site between P1 and J23 in K_{neu}_free_1.

File Name: Supplementary Movie 4

Description: The structural changes of AARA induced by K^{+} ion binding events in $K_{0.3}$_free_2.

File Name: Supplementary Movie 5

Description: The structural correlation between J23 and $C_{J12\text{-}P1}$ in $K_{0.3}$_free_2.

File Name: Supplementary Movie 6

Description: The transition of stacking pattern of base A24 (involving A73 stacked below it) between two bases (C54 and G72) in $K_{0.3}$_free_2.

Supplementary References

1. Gabb, H. A., Sanghani, S. R., Robert, C. H. and Prévost, C. Finding and visualizing nucleic acid base stacking. *J. Mol. Graph.* **14**, 6–11 (1996).
2. Park, S., Bardhan, J. P., Roux, B. and Makowski, L. Simulated x-ray scattering of protein solutions using explicit-solvent models. *J. Chem. Phys.* **130**, 134114 (2009).
3. Nguyen, H. T. , Pabit, S. A. , Meisburger, S. P. , Pollack, L. and Case, D. A. Accurate small and wide angle x-ray scattering profiles from atomic models of proteins and nucleic acids. *J. Chem. Phys.* **141**, 22D508 (2014).
4. Serganov, A., Yuan, Y. R., Pikovskaya, O., Polonskaia, A., Malinina, L., Phan, A. T., Hobartner, C., Micura, R., Breaker, R. R. and Patel, D. J. Structural basis for discriminative regulation of gene expression by adenine- and guanine-sensing mRNAs. *Chem. Biol.* **11**, 1729–1741 (2004).
5. Zhang, J. and Ferré-D'Amaré, A. R. Dramatic improvement of crystals of large RNAs by cation replacement and dehydration. *Structure* **22**, 1363–1371 (2014).
6. Stagno, J. R., Liu, Y., Bhandari, Y. R., Conrad, C. E., Panja, S., Swain, M., Fan, L., Nelson, G., Li, C., Wendel, D. R., White, T. A., Coe, J. D., Wiedorn, M. O., Knoska, J., Oberthuer, D., Tuckey, R. A., Yu, P., Dyba, M., Tarasov, S. G., Weierstall, U., Grant, T. D., Schwieters, C. D., Zhang, J., Ferré-D'Amaré, A. R., Fromme, P., Draper, D. E., Liang, M., Hunter, M. S., Boutet, S., Tan, K., Zuo, X., Ji, X., Barty, A., Zatsepin, N. A., Chapman, H. N., Spence, J. C. H., Woodson, S. A. and Wang, Y.-X. Structures of riboswitch RNA reaction states by mix-and-inject XFEL serial crystallography. *Nature* **541**, 242–246 (2017).
