# Supplementary material for: Potential effects of metal ion induced two-state allostery on the regulatory mechanism of add adenine riboswitch
Source: Commun Biol. 2022 Oct 22;5:1120. doi: 10.1038/s42003-022-04096-z (PMC9588036; doi:10.1038/s42003-022-04096-z)
Supplement: Supplementary file 3 — Description of Additional Supplementary Data [file 42003_2022_4096_MOESM3_ESM.docx]

**Description of Additional Supplementary Files**

**File name:** Supplementary Movie 1

**Description:** The first principal component for Mg0.3_free_1 shows that the Mg2+ ion bound at CJ12-P1 site fluctuates with the surrounding structure.

**File name:** Supplementary Movie 2

**Description:** The rapid exchange phenomenon of Mg2+ ions at CJ12-P1 site in Mg0.3_free_1. The chelated water molecules around Mg2+ ions and the structure near the binding site are depicted with licorice drawing method.

**File name:** Supplementary Movie 3

**Description:** The rapid exchange phenomenon of K+ ions at the binding site between P1 and J23 in Kneu_free_1.

**File name:** Supplementary Movie 4

**Description:** The structural changes of AARA induced by K+ ion binding events in K0.3_free_2.

**File name:** Supplementary Movie 5

**Description:** The structural correlation between J23 and CJ12-P1 in K0.3_free_2.

**File name:** Supplementary Movie 6

**Description:** The transition of stacking pattern of base A24 (involving A73 stacked below it) between two bases (C54 and G72) in K0.3_free_2.

**File name:** Supplementary Data 1

**Description:** The initial and final PDB structure files of the full system and simulation input files.

**File name:** Supplementary Data 2

**Description:** All source data underlying the graphs and charts presented in the main figures.
